# Evaluation of the endoplasmic reticulum-stress response in eIF2B-mutated lymphocytes and lymphoblasts from CACH/VWM patients

**DOI:** 10.1186/1471-2377-10-94

**Published:** 2010-10-19

**Authors:** Laetitia Horzinski, Liraz Kantor, Aurélia Huyghe, Raphael Schiffmann, Orna Elroy-Stein, Odile Boespflug-Tanguy, Anne Fogli

**Affiliations:** 1 INSERM U931-CNRS 6247-Génétique, Reproduction et Développement (GReD)-Faculté de Médecine, Clermont-Ferrand, France; 2 Université Clermont1, UFR Médecine, Clermont-Ferrand, France; 3 Department of Cell Research and Immunology, George S. Wise Faculty of Life Sciences, Tel-Aviv University, Tel-Aviv, Israel; 4 Institute of Metabolic Disease, Baylor Research Institute, Dallas, Texas, USA; 5 CHU de Clermont-Ferrand, Service de Génétique Médicale, Centre de Référence Leucodystrophies, Hôpital Hôtel-Dieu, F-63058 Clermont-Ferrand, France; 6 CHU de Clermont-Ferrand, Service de Biochimie Médicale et Biologie Moléculaire, Clermont-Ferrand, France

## Abstract

**Background:**

Eukaryotic translation initiation factor 2B (eIF2B), a guanine nucleotide exchange factor (GEF) and a key regulator of translation initiation under normal and stress conditions, causes an autosomal recessive leukodystrophy of a wide clinical spectrum. EBV-immortalised lymphocytes (EIL) from eIF2B-mutated patients exhibit a decrease in eIF2B GEF activity. eIF2B-mutated primary fibroblasts have a hyper-induction of activating transcription factor 4 (ATF4) which is involved in the protective unfolded protein response (UPR), also known as the ER-stress response. We tested the hypothesis that EIL from eIF2B-mutated patients also exhibit a heightened ER-stress response.

**Methods:**

We used thapsigargin as an ER-stress agent and looked at polysomal profiles, rate of protein synthesis, translational activation of *ATF4*, and transcriptional induction of stress-specific mRNAs (*ATF4, CHOP, ASNS, GRP78*) in normal and eIF2B-mutated EIL. We also compared the level of stress-specific mRNAs between EIL and primary lymphocytes (PL).

**Results:**

Despite the low eIF2B GEF activity in the 12 eIF2B-mutated EIL cell lines tested (range 40-70% of normal), these cell lines did not differ from normal EIL in their ATF4-mediated ER-stress response. The absence of hyper-induction of ATF4-mediated ER-stress response in eIF2B-mutated EIL in contrast to primary fibroblasts is not related to their transformation by EBV. Indeed, PL exhibited a higher induction of the stress-specific mRNAs in comparison to EIL, but no hyper-induction of the UPR was noticed in the eIF2B-mutated cell lines in comparison to controls.

**Conclusions:**

Taken together with work of others, our results demonstrate the absence of a major difference in ER-stress response between controls and eIF2B-mutated cells. Therefore, components of the ER-stress response cannot be used as discriminantory markers in eIF2B-related disorders.

## Background

The eukaryotic initiation factor 2B (eIF2B) is a ubiquitously expressed protein with guanine nucleotide exchange factor (GEF) activity that is involved in the translation initiation step by activating the eIF2 factor. Mutations in any one of the *EIF2B1-5 *genes [Swiss-Prot: Q14232, P49770, Q9NR50, Q9UI10, Q13144], encoding the five eIF2B subunits, have been initially described in an autosomal recessive form of inherited disorders, the childhood ataxia with central hypomyelination (CACH)/Vanishing white matter (VWM) syndrome [1-4]. This leukoencephalopathy is characterized in infants by a progressive neurological deterioration exacerbated by episodes of stress and a cerebrospinal fluid (CSF)-like signal intensity of the WM on brain magnetic resonance imaging (MRI) [1,2]. The use of a typical MRI pattern to select patients with undetermined leukodystrophies for *EIF2B1-5 *genes analysis demonstrated the wide clinical *spectrum *of eIF2B-mutated patients from congenital and rapidly lethal forms to slowly progressive or even asymptomatic adult forms associated in some cases with ovarian failure [5,6]. The need for a reliable and quick diagnostic marker, useful to select patients eligible for *EIF2B1-5 *sequencing became obvious.

eIF2B is a key regulator of the translation initiation step by its nucleotide guanine exchange activity (GEF activity) that converts the eIF2 factor from an inactive GDP-bound form to an active GTP-bound form followed by initiation of the first step of the translation process. We previously reported a decrease eIF2B GEF activity in Epstein-Barr virus (EBV)-immortalised lymphocytes (EIL, or lymphoblasts) from patients with eIF2B-related disorder that seemed to correlate with disease severity [7]. eIF2B GEF activity is subjected to diverse modes of regulation. One of the inhibitory mechanisms is mediated by phosphorylation of Ser 51 of eIF2α subunit by four specific kinases (PERK, GCN2, PKR and HRI), each of which is activated by specific stress conditions including endoplasmic reticulum (ER)-stress [8]. eIF2 phosphorylation mediates the inhibition of eIF2B, leading to reduction of global translation initiation which is accompanied by translational activation of specific sub-class of mRNAs encoding rescue proteins. One example is translational induction of the mRNA encoding activating transcription factor 4 (ATF4) under stress conditions.

ATF4-mediated heightened stress response was demonstrated in several eIF2B-mutated models [9-16]. In yeast, it has been shown that eIF2B mutations induce a decrease of eIF2B GEF activity, leading to the translation activation of *GCN4 *mRNA, the yeast homolog of ATF4 involved in stress regulation [10,11]. Later on, heightened ER-stress response mediated by hyper-induction of ATF4 was observed in primary fibroblasts from eIF2B-mutated patients [9], and in rat oligodendroglial-derived cells expressing mutated human *EIF2B5 *gene [12]. Similarly, increased levels of ER-stress markers were detected in cerebral white matter from eIF2B-mutated patients [13,14]. Surprisingly, although eIF2B mutations render the mutated cells hyper-sensitive to stress, eIF2B GEF activity was not significantly decreased in most of the above mentioned cell types. The only human cell lines exhibiting a measurable decrease of eIF2B GEF activity were lymphoblasts from eIF2B-mutated patients [7,16]. A single study reported stress-related differences between control and eIF2B-mutated patient's EIL: mutated EIL lost their ability to respond to heat stress by eIF2α phosphorylation, suggesting a lack of inhibition of eIF2B GEF activity under stress conditions [17] leading to a potentially disturbed cellular response.

In the current study, we tested the hypothesis that EIL from eIF2B-mutated patients would also express a heightened ER-stress response that would be useful in understanding the mechanism of the disease. For this purpose, we compared the effect of ER-stress on eIF2B-mutated and control EIL by looking at i) polysomal profiles; ii) rate of protein synthesis; iii) translational induction of *ATF4 *mRNA; and iv) transcriptional induction of mRNA encoding ATF4, C/EBP-Homologous Protein (CHOP), Asparagine synthetase (ASNS) and Glucose-Regulated protein 78 kDa (GRP78).

## Methods

### Selection of patients and generation of lymphoblasts

Fourteen eIF2B-mutated patients from 13 affected families were selected according to age at disease onset (from age 0.8 to 16 years) and type of eIF2B mutations (Table 1) [16], in comparison to nine age- and sex-matched healthy patients used as controls. An Institutional Review Board of the participating centers (Comité de Protection des Personnes Sud-Est VI, 2009-A00188-49) approved the use of human subjects for this study. A written informed consent was obtained from all patients.

**Table 1:** List of the eIF2B-mutated patients selected for this study

Patients' number	DNA mutation (*mutated gene*)	Protein mutation	GEF activity (%)
569-3	c.338G > A/c.766-1G > A (*EIF2B5*)	p.Arg113His/p.256_281del	40 ± 2

571-1	c.166T > G/c.944G > A (*EIF2B5*)	p.Phe56Val/p.Arg315His	40 ± 3

357-2	c.406C > T/c.1015C > T(*EIF2B5*)	p.Arg136Cys/p.Arg339Trp	44.5 ± 4.5

1078-1	c.407G > A/c.407G > A (*EIF2B5*)	p.Arg136His/p.Arg136His	49.6 ± 5.7

432-2	c.271A > G/c.1015C > T (*EIF2B5*)	p.Thr91Ala/p.Arg339Trp	53.9 ± 0,.9

522-1	c.47C > A/c.338G > A (*EIF2B5*)	p.Ala16Asp/p.Arg113His	54 ± 6

648-1*	c.638A > G/c.638A > G (*EIF2B2*)	p.Glu213Gly/p.Glu213Gly	59 ± 1

648-2*	c.638A > G/c.638A > G (*EIF2B2*)	p.Glu213Gly/p.Glu213Gly	64 ± 4

1014-1	c.338G > A/c.583C > T(*EIF2B5*)	p.Arg113His/p.Arg195Cys	68 ± 4

338-1	c.338G > A/c.338G > A (*EIF2B5*)	p.Arg113His/p.Arg113His	75.2 ± 1.5

370-2	c.338G > A/c.584G > A(*EIF2B5*)	p.Arg113His/p.Arg195His	77 ± 2.5

630-1	c.338G > A/c.338G > A (*EIF2B5*)	p.Arg113His/p.Arg113His	77.5 ± 2.5

1241-1*	c.338G > A/c.338G > A (*EIF2B5*)	p.Arg113His/p.Arg113His	NA

807-1*	c.338G > A/c.338G > A (*EIF2B5*)	p.Arg113His/p.Arg113His	67 ± 4.3

The clinical and genetic status of these patients has previously been described [16]. Patients' lymphoblasts were used to quantify the protein and transcript expression of the ER-stress genes in comparison to associated controls. * Patients for whom ER-stress responses were compared between lymphoblasts and lymphocytes.

NA: not available.

Table adapted from Horzinski et al. [16]

Patients' lymphocytes (PL) were immortalized with the Epstein-Barr virus (EBV) according to classical procedures [18].

Twelve eIF2B-mutated EIL were used to compare the protein and transcript expression after ER-stress, in comparison to six associated control EIL. PL and EIL from four eIF2B-mutated patients (Table 1) were used to analyze the impact of PL immortalization on the transcript ER-stress response, in comparison to PL and EIL from three healthy controls (C1, C2, 1283-1).

### eIF2B GEF activity

The GEF activity of eIF2B was measured in triplicate as previously described [7].

### ER-stress induction

Patients' EIL and PL were incubated with 0.5, 1 or 2 μg/ml Thapsigargin (Tg) for 4 h or 1.5 h at 37°C, harvested just after the treatment or submitted to recovery for 1 h, 3 h, 6 h, 12 h, 15 h, 18 h or 24 h under normal.

### Global protein synthesis rate and polysomal profiles

Global protein synthesis rates were determined as described previously [19] by using EIL incubated in the absence or presence of 2 μg/ml Tg for 1 hour for labeling with [^35^S]L-methionine/[^35^S]L-cysteine mix for 20 min. Polysomal profiles were performed on 0-50% sucrose gradients according to Sivan *et al. *[19] using 1 × 10^7 ^EIL per gradient.

### Immunodetection of ATF4

Western blot analysis of ATF4 was performed as previously described [9] using EIL, incubated in the absence or presence of 2 μg/ml Tg for 4 hours.

### Quantitative Real-Time Polymerase Chain Reaction (QRT-PCR)

Total RNA was extracted from pellets of 15.10^6 ^cells using TRIZOL solution (Invitrogen Life Technology) according to classical procedures. One μg RNA was subjected to RT, performed in duplicate, with 0.5 mM dNTP, 0.025 μg/μl oligodT, 1× First Strand Buffer, 10 mM DTT, 40 U Rnase Out and 200 U SuperScript™III Rnase H-Reverse Transcriptase (Invitrogen) at 42°C during 1 h then 70°C during 15 min. Quantitations of *ATF4*, *CHOP*, *ASNS*, *GRP78 *and β*2-microglobulin *(*B2M*) mRNA levels were carried-out in duplicate on Light Cycler (Roche) using the SybrGreen technology and the specific set of primers: ATF4-F: 5'-CCCCTTCACCTTCTTACAACC-3'; ATF4-R: 5'-GGGCTCATACAGATGCCACAT-3'; CHOP-F: 5'-CAGAACCAGCAGAGGTCACA 3'; CHOP-R: 5'-AGCTGTGCCACTTTCCTTTC-3'; GRP78-F: 5'-GGTTGATTATCAGAAGCTGTAG-3'; GRP78-R: 5'-CGTATGGTGCTGCTGTCCAGG-3'; ASNS-F: 5'-ATCACTGTCGGGATGAACCC-3'; ASNS-R: 5'-CTTCAACAGAGTGGCAGCAA-3'; B2M-F: 5'-TGTCTTTCAGCAAGGACTGG-3'; B2M-R: 5'-TTCTCTGCTCCCCACCTCTA-3'. One μl of 1:10 cDNA dilution was amplified with 1× LightCycler FastStart Reaction Mix (Roche), 3 mM MgCl_2 _and 0.5 mM of each primer in a final volume of 15 μl. The program included an initial denaturation (95°C, 10 min) followed by 45 cycles of 95°C for 10 seconds, 60°C for 30 seconds and 72°C for 30 seconds. The cycle of threshold value (Ct) was used to calculate the relative expression of the genes of interest *ATF4*, *CHOP*, *ASNS *or *GRP78*, normalized to *β2M*.

## Results

### Effects of eIF2B mutations on eIF2B GEF activity and on the translation rates in lymphoblasts

eIF2B GEF activities measured in the 12 eIF2B-mutated EIL were significantly lower (range 40%-78% of normal) in comparison to the six controls. However, the translational machineries of both normal and mutated EIL were inhibited to the same extent by the presence of the ER-stress agent, as judged by the incorporation rate of [^35^S]L-methionine/[^35^S]L-cysteine which was reduced to 40 ± 5% or 39 ± 3% for patients or control EIL, respectively; and by the similar decrease in heavy polysomes (data not shown).

These results demonstrate the absence of significant effect of eIF2B mutations on the intensity of the ER-stress response despite decrease eIF2B GEF activity.

### Effects of eIF2B mutations on the ER-stress response mediated by ATF4 in lymphoblasts

**Figure 1:**
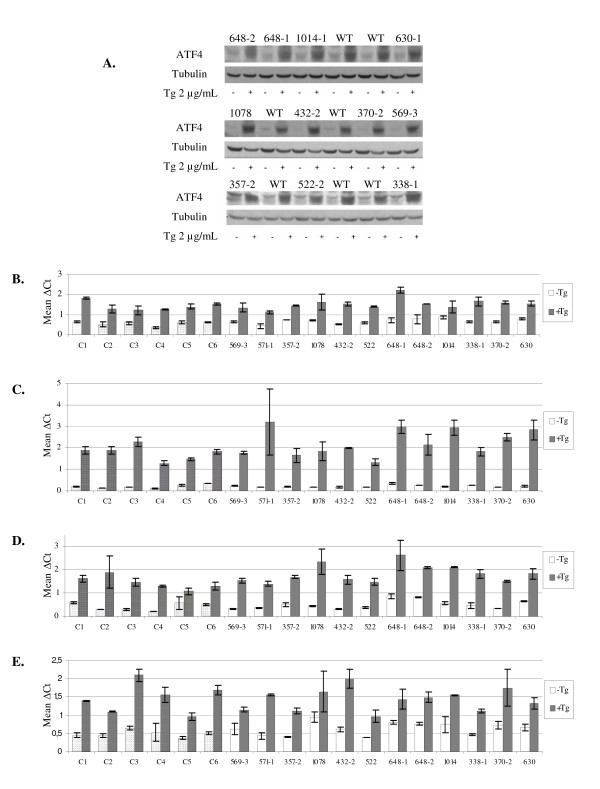
**Expression level of ATF4 protein and four mRNA markers of the ER-stress response in lymphoblasts from eIF2B-mutated patients**. **(A) **ATF4 protein induction (Western-blot); WT: control individuals; (+), (-): with or without 4 h thapsigargin (Tg) treatment. **(B-E) **Expression levels of *ATF4 *(**B**), *CHOP *(**C**), *ASNS *(**D**) and *GRP78 *(**E**) mRNA determined by quantitative real-time polymerase chain reaction; from eIF2B-mutated patients (non-stippled bars), control individuals (stippled bars); under normal conditions (white bars) or following 4 h Tg treatment (grey bars); mean ΔCt values: mean Ct values of genes of interest corrected for the Ct value of the *β2M *gene.

Next, we evaluated the impact of stress response by measuring ATF4 protein level in EIL incubated for 4 hours in the presence or absence of 2 μg/ml Tg. As expected, an increase in ATF4 protein expression was found under ER-stress conditions (Figure 1A). However, no differences were observed between controls and eIF2B-mutated EIL, in ATF4 basal levels and ATF4 induced upon ER-stress. In the Tg-treated EIL, an expected significant increase in the level of *ATF4*, *CHOP*, *ASNS *and *GRP78 *mRNAs was also observed, which was similar in normal and eIF2B-mutated EIL, confirming the comparable activation of the ER-stress response in the two cell types (Figure 1B-E). An inter-individual variability was high in stressed as well as non-stressed cells, and no significant differences between controls and patients were observed. Moreover, we did not find any correlation between the rate of eIF2B GEF activity and the level of ATF4 translational induction or transcriptional activation of *ATF4*, *CHOP*, *ASNS *and *GRP78*. Likewise, other ER-stress conditions (0.5 or 1 μg/ml Tg for 4 h or 1.5 h) before or after different recovery times (1 h, 3 h, 6 h, 12 h, 15 h, 18 h and 24 h) did not result in any significant difference in the intensity of the stress response between eIF2B-mutated and non-mutated EIL (data not shown).

### Effect of the EBV immortalization on the ER-stress response mediated by ATF4

**Figure 2:**
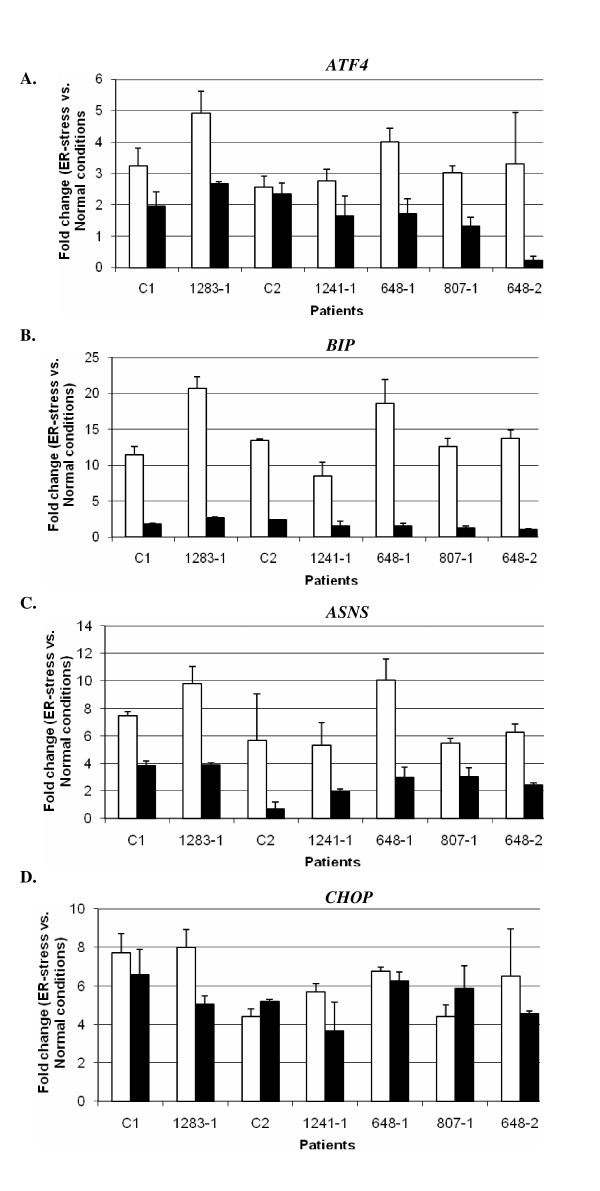
**Effect of the lymphocytes EBV-transformation on the expression level of the four mRNA markers of the ER-stress response**. Thapsigargin (incubation with Thapsigargin (Tg) at 2 μg/ml for 4 h) was used to induce ER-stress in lymphoblastoid cell lines (EIL, black bars) and primary lymphocytes (PL, white bars) from control (C1, C2 and 1283-1) and eIF2B-mutated (1241-1, 807-1, 648-1 and 648-2) patients. Expression levels of *ATF4 *(**A**), *GRP78 *(**B**), *ASNS *(**C**) *and CHOP *(**D**) mRNA were determined by quantitative real-time polymerase chain reaction. The level of each transcript was calculated using the 2^(-ΔΔCt) method, followed by normalization of the primary Ct data to beta2M level. Results are shown as fold-change between ER-stress (Tg incubation) compared to normal conditions.

As primary eIF2B-mutated fibroblasts exhibit heightened ER-stress response mediated by hyper-induction of ATF4 [6], a possible explanation for the lack of difference between eIF2B-mutated and normal EIL on the ER-stress activation could be the immortalization of the PL by EBV. Therefore, we evaluated the impact of EBV immortalization on ER-stress response by comparing EIL and PL from four eIF2B-mutated patients (Table 1) and three healthy individuals using the same stress induction procedure. We found an expected increase in the level of *ATF4*, *CHOP*, *ASNS *and *GRP78 *mRNA in all the cells, but it was more profound in PL (Figure 2). No significant differences were found in the activation of ER-stress response between eIF2B-mutated and control PL, as was demonstrated in EIL (Figure 1), and extended here to PL (Figure 2). Therefore, modifications induced by EBV-immortalization of PL are not responsible for the absence of differential ER-stress response between eIF2B-mutated and control cells.

Together, these results show that the eIF2B-mutated EIL or PL do not differ from normal cells in their ATF4-mediated ER-stress response induced by Tg despite the low eIF2B GEF activity found in mutated EIL.

## Discussion

In the present study, we evaluated the ER-stress response pathway mediated by ATF4 in eIF2B-mutated EIL in comparison to control cells. Our results confirmed that eIF2B activity was reduced in mutated EIL, but without significant hyperactivation of the UPR pathway. However, an abnormal heightened stress response was identified earlier in patients' brains [13] and in primary fibroblasts [9]. Cells from the same four patients (1048-1, 432-2, 522-1 and 1241-1) were analysed in this study and in the Kantor *et al. *paper [9]. We previously demonstrated that transformation of primary fibroblasts from eIF2B-mutated patients by SV40 large-T antigen abolished the heightened stress response identified in eIF2B-mutated compared to normal fibroblasts [9]. Accordingly, we anticipated that EBV-transformation may abolish the possible hyper-sensitivity of eIF2B-mutated lymphocytes to ER-stress agents. EBV-transformation activates cyclin D2 and additional proteins involved in cell cycle progression, including E2Fs, pRb and BCL-2, reflecting cellular resistance to apoptosis [20]. Moreover, lymphoblasts have a modified metabolism towards an anti-apoptotic phenotype, exerted by EBV antigen 3A (*EBNA3A*)-mediated induction of protective chaperones and co-chaperones such as Hsp70 [21]. In contrast to our prediction, we found that EBV-transformation of PL is not responsible for the absence of the anticipated heightened ER-stress response of eIF2B-mutated compared to control PL. However, EBV-transformation did reduce the strength of the ER-stress response in both cell types, as demonstrated by the higher induction of ATF4 and GRP78 in PL compared to EIL (Figure 2).

Our data are in agreement with results of van Kollenburg *et al *[17], who studied stress-related differences between eIF2B-mutated and normal lymphoblasts after heat shock and who found a lesser increase in eIF2α phosphorylation in patients' EIL in response to the heat-shock. Although the experimental variables involved in the current study do not allow to reproducibly pick subtle changes in eIF2α phosphorylation level, even a slight eIF2α hypo-phosphorylation response may explain the absence of heightened ER-stress response in eIF2B-mutated PL and EIL used here. None of the mutations studied here correspond to mutations in yeast previously shown to render eIF2B resistant to the effects of eIF2α phosphorylation [10,11,15]. Immortalization of mouse fibroblasts was found to increase eIF2B expression leading to increased GEF activity and to reduced sensitivity to eIF2 phosphorylation [22]. We therefore hypothesize that eIF2 expression is increased in EIL in comparison to PL, making i) the GEF assay more robust in EIL in which differences in GEF activity between control and eIF2B-mutated patients are more easily discerned, and ii) EIL are less sensitive to increased eIF2 phosphorylation following UPR in comparison to PL [22]. Further analyses are needed to determine if the normal ER-stress response found in eIF2B-mutated EIL despite a low eIF2B GEF activity is also a trait of other non-transformed, yet actively dividing eIF2B-mutated cells during the course of development. This could contribute to the observation of a cell fate impairment restricted to glial cell development in the large majority of eIF2B-mutated patients [23].

## Conclusions

Lymphoblasts generated from patients' blood samples demonstrated a decrease in eIF2B GEF activity, a test already proposed for the diagnostic screening and prognosis of eIF2B-related disorders. However, measurement of EIL and PL ER-stress response did not show any differences between eIF2B-mutated and control cells and therefore is not useful as a supplementary test to improve diagnosis.

## Competing interests

The authors declare that they have no competing interests.

## Authors' contributions

LH performed stress assay and carried out the mRNA analysis of EIL. LK performed stress assay and carried out the protein analysis of EIL. AH participated to the cell culture. RS performed the clinical diagnosis of eIF2B-mutated patients, provided fibroblasts and helped to draft the manuscript. OES participated in the design of the study and helped to draft the manuscript. OBT participated in the design of the study, performed the clinical diagnosis of eIF2B-mutated patients, provided fibroblasts and helped to draft the manuscript. AF performed stress assay and carried out the mRNA analysis of PL in comparison to EIL, participated in the design of the study and helped to draft the manuscript.

## Pre-publication history

The pre-publication history for this paper can be accessed here:

http://www.biomedcentral.com/1471-2377/10/94/prepub
